# The Development of a CRISPR-FnCpf1 System for Large-Fragment Deletion and Multiplex Gene Editing in *Acinetobacter baumannii*

**DOI:** 10.3390/cimb46010037

**Published:** 2024-01-05

**Authors:** Shuai Wang, Yue Ding, Hua Rong, Yu Wang

**Affiliations:** 1 College of Bioscience and Bioengineering, Jiangxi Agricultural University, Nanchang 330045, China; wangshuai98125@163.com (S.W.); dyueyue0712@163.com (Y.D.); 2 Nanchang City Key Laboratory of Animal Virus and Genetic Engineering, Nanchang 330045, China

**Keywords:** *Acinetobacter baumannii*, CRISPR-FnCpf1, large-fragment deletion, multiplex gene editing

## Abstract

*Acinetobacter baumannii* is a low-GC-content Gram-negative opportunistic pathogen that poses a serious global public health threat. Convenient and rapid genetic manipulation is beneficial for elucidating its pathogenic mechanisms and developing novel therapeutic methods. In this study, we report a new CRISPR-FnCpf1-based two-plasmid system for versatile and precise genome editing in *A. baumannii*. After identification, this new system prefers to recognize the 5′-TTN-3′ (N = A, T, C or G) and the 5′-CTV-3′ (V = A, C or G) protospacer-adjacent motif (PAM) sequence and utilize the spacer with lengths ranging from 19 to 25 nt. In direct comparison with the existing CRISPR-Cas9 system, it exhibits approximately four times the targetable range in *A. baumannii*. Moreover, by employing a tandem dual crRNA expression cassette, the new system can perform large-fragment deletion and simultaneous multiple gene editing, which is difficult to achieve via CRISPR-Cas9. Therefore, the new system is valuable and can greatly expand the genome editing toolbox of *A. baumannii*.

## 1. Introduction

*Acinetobacter baumannii* is a low-GC-content Gram-negative opportunistic pathogen, causing a wide variety of hospital-acquired infections such as wound infection, bacteremia, urinary tract infection, meningitis, and ventilator-associated pneumonia [1]. Due to an increase in the prevalence of multidrug-resistant *A. baumannii* in recent years, it has been considered one of the most difficult-to-treat pathogens in hospital settings, particularly in the intensive care unit (ICU). The mortality rate among ICU patients infected by *A. baumannii* is as high as 45–60%, which may increase to over 80% when multiple drug-resistant (MDR) or extensively drug-resistant (XDR) specimens among these pathogens are involved [2,3,4]. However, therapeutic means against MDR and XDR *A. baumannii* infections are very limited.

Convenient and efficient genetic manipulation tools are indispensable for the development of new therapeutic means and identification of drug resistance and pathogenicity mechanisms. Despite the establishment of the CRISPR-spCas9 (hereinafter referred to as Cas9)-based genome editing method in *A. baumannii* [5], certain genetic manipulations, such as large-fragment deletion and multiplex gene editing, remain constrained.

Cpf1, also known as Cas12a, is a type V-A CRISPR-Cas RNA-guided endonuclease [6]. Currently, three CRISPR-Cpf1 systems (AsCpf1, LbCpf1, and FnCpf1) are available for genome editing in diverse organisms [7,8,9,10]. The result of a plasmid depletion assay showed that, compared to AsCpf1 and LbCpf1 utilizing the 5′-TTTN-3′ as a protospacer-adjacent motif (PAM) sequence, FnCpf1 recognized a shorter DNA sequence, the 5′-TTN-3′, as PAM. This characteristic grants FnCpf1 a significantly broader potential targeting range within genome editing [11]. Several distinct features of CRISPR-Cpf1 compared with those of CRISPR-Cas9 lead to the advantages of using CRISPR-Cpf1 for genome editing. First, Cpf1 recognizes a T-rich PAM sequence, distinct from the G-rich PAM sequence recognized by Cas9, and thereby, Cpf1 possesses more editable sites in organisms with AT-rich genomes [12], such as *A. baumannii*. Second, Cpf1 cleaves target DNA with the sites that are distal to PAM and creates a sticky DNA end [11], whereas Cas9 cleaves target DNA with the sites that are proximal to PAM by generating a blunt DNA end [13]. The sticky end may improve the recombination repair efficiency. Third, Cpf1 is guided by a short single crRNA (~44 nt) and does not require a tracrRNA, resulting in a shorter guide RNA sequence than the crRNA-tracrRNA chimera (sgRNA, ~96 nt) used by Cas9 [14]. The small size offers simpler and cheaper guide RNA production. Fourth, different from Cas9 DNA nuclease, Cpf1 is a dual nuclease that not only cleaves target DNA but also processes its own crRNA array, which is beneficial to multiplex genome editing [15].

In this study, we develop a new CRISPR-FnCpf1-based two-plasmid system, which could achieve versatile and precise genome editing in various strains of *A. baumannii*. The preferred PAM sequence and the spacer length by FnCpf1 in *A. baumannii* are systematically identified. Moreover, we compare the editing efficiency of Cas9 and FnCpf1 in *A. baumannii*. Additionally, this tool is successfully applied to delete large fragments and edit multiple genes simultaneously using a tandem crRNA array in *A. baumannii*. Collectively, the newly developed CRISPR-FnCpf1 system is valuable for precise genetic engineering and can greatly expand the genome editing toolbox of *A. baumannii*.

## 2. Materials and Methods

### 2.1. Bacterial Strains, Plasmids, Primers, Culture Conditions, and Antibiotics

The bacterial strains used for genome editing experiments in this study were the wild-type *A. baumannii* ATCC 17978, ATCC 19606, clinically isolated ABH6, and their derivative strains. The commercial *E. coli* Top10 competent cells was purchased from Tolo Biotech (Shanghai, China), and was used as cloning host. All of the plasmids used in this study are listed in Table S1. The *J23119*-crRNA scaffold-double *Bsa*I-HDV-*metZWV* expression cassette was synthesized by Genewiz (Suzhou, China). The primers and single-stranded donor oligonucleotides (ssODN) donors used in this study were synthesized either by Genewiz (Suzhou, China) or by Tsingke (Changsha, China), and their sequences are listed in Table S2. The *E. coli* Top10 strain and all the *A. baumannii* strains were cultured in lysogeny broth (LB) medium (per liter, 5 g yeast extract, 10 g tryptone, 10 g NaCl [pH 7.2–7.4]) at 37 °C, unless indicated otherwise. When appropriate, antibiotics were added at the following concentrations: for *E. coli* Top10, 60 µg/mL apramycin, 50 µg/mL kanamycin, 50 µg/mL spectinomycin; for *A. baumannii*, 50–100 µg/mL apramycin, 50 µg/mL kanamycin, 100 µg/mL spectinomycin.

### 2.2. Plasmid Construction

The pFnCpfAb-apr plasmid was constructed as follows. The FnCpf1 coding fragment was amplified by PCR from the pKOBEG-rpsL-FnCpf1 plasmid (Figure S1) and inserted to replace the Cas9 coding fragment of the pET-tac-Cas9 plasmid, resulting in the pET-tac-FnCpf1 plasmid. The FnCpf1-sacB fragment was PCR-amplified from the aforementioned pET-tac-FnCpf1 plasmid and cloned into the *Bam*HI site of the pAT04-apr plasmid by Gibson assembly, resulting in the final pFnCpfAb-apr plasmid.

The pCrAb-km plasmid was constructed by replacing the sgRNA expression cassette of the pSGAb-km plasmid with the crRNA expression cassette between the *Hin*dIII and the *Eco*RI sites. The pCrAb-spe plasmid was generated by replacing the kanamycin resistance gene of the pCrAb-km plasmid with the spectinomycin resistance gene via Gibson assembly.

### 2.3. Preparation of Electrocompetent Cells

Preparation of *A. baumannii* electrocompetent cells was performed according to previously described procedures [5]. Briefly, for the wild-type strain, 500 µL of overnight culture from a freshly streaked single colony was inoculated into 50 mL of LB broth and incubated with shaking at 37 °C. When the cells reached an optical density at 600 nm (OD_600_) of 0.6, the culture was immediately chilled on ice for 10 min. Then, the cells were harvested by centrifugation at 4 °C for 5 min at 4000× *g*. The supernatant was discarded, and the cell pellet was washed once with sterile ice-cold Milli-Q water and once with sterile ice-cold 10% glycerol (*v*/*v*). Finally, the cell pellet was resuspended in 500 μL of sterile ice-cold 10% glycerol (*v*/*v*). Then, 50 μL single-use aliquots were flash-frozen in liquid nitrogen and stored at −80 °C until use.

For the pFnCpfAb-apr-harboring strain, 500 µL of overnight culture of transformants was inoculated into 50 mL of LB broth with apramycin and incubated at 37 °C with shaking until an OD_600_ value of 0.1–0.15 (approximately one hour) was reached. At this point, a final concentration of 1 mM IPTG was added. The culture was further incubated for no less than 2 h to induce the expression of RecAb recombination proteins and FnCpf1 nuclease. The same protocol was used for preparation of the electrocompetent cells as in the method described above. As a crucial point of this assay, the pFnCpfAb-apr-harboring *A. baumannii* electrocompetent cells cannot be stored frozen. The utilization of the frozen pFnCpfAb-apr-harboring *A. baumannii* electrocompetent cells would significantly decrease the efficiency of genome editing.

### 2.4. Electrocompetent

No more than 5 µL of plasmid or the plasmid/donor mixture was added into the 50 µL of electrocompetent cells. For the frozen wild-type electrocompetent cells, the cells should be thawed on ice for several minutes and suspended well by carefully flicking the tubes prior to adding DNA. The DNA/cell mixture was transferred into a pre-chilled 2 mm gap electroporation cuvette and electroporated at 2.5 kV, 200 Ω, and 25 µF. All electroporation was conducted on a Bio-Rad Gene Pulser. The time constant for the pulse was approximately 5 ms. After being pulsed, the cells were diluted with 1 mL of antibiotic-free LB broth and incubated at 37 °C for 1–2 h before being plated onto LB agar plates supplemented with the corresponding antibiotics. The plates were incubated at 37 °C until single colonies were clearly discernable.

### 2.5. Spacer Insertion

We selected a 19–25 bp spacer sequence located downstream of the PAM site in the target gene of *A. baumannii*. Two oligos were designed and synthesized with overhang sequences as “Spacer-F: 5′-agatNNNNNNNNNNNNNNNNNNNN (forward)-3′” and “Spacer-R: 5′-ggccNNNNNNNNNNNNNNNNNNNN (backward)-3′”. Then, the two oligos were phosphorylated using T4 PNK in a system of 25 μL (Spacer-F (10 μM) 5 μL, Spacer-R (10 μM) 5 μL, 10 × T4 DNA Ligase buffer 2.5 μL, T4 PNK 0.5 μL, ddH_2_O 12 μL). After phosphorylation, 0.5 μL of 2.5 M sodium chloride buffer was added into the reaction product and mixed gently. The tube was incubated at 95 °C for 3 min and then slowly cooled down to room temperature using a thermocycler set to decrease 1 °C per 10 s. The annealed oligos were 20-fold diluted with sterile ddH_2_O and then inserted into the pCrAb plasmid using the Golden Gate assembly method. Finally, the assembly product was transformed into 100 μL of *E. coli* Top10 competent cells according to manufacturer’s instructions. The *E. coli* cells were plated onto an LB agar plate supplemented with the corresponding antibiotic and incubated at 37 °C overnight. The correct clone insertion was verified by colony PCR with the primers of Spacer-F and M13R and by sequencing with the M13R universal primer.

### 2.6. Genome Editing with CRISPR-FnCpf1

Approximately 50 ng of the pFnCpfAb-apr plasmid was electroporated into the wild-type strain as in the method described above. The cells were plated onto an LB agar plate supplemented with 50 μg/mL apramycin and incubated at 37 °C overnight. A single colony was picked up and made into the IPTG-induced pFnCpfAb-apr-harboring *A. baumannii* electrocompetent cells. Then, 1 μg (or less) of spacer-introduced pSGAb-km plasmid and 3 μL of 100 μM ssODN donor were co-electroporated into the recipient cells as in the method described above. The cells were plated onto an LB agar plate supplemented with 50 μg/mL apramycin and 50 μg/mL kanamycin. The plate was incubated at 37 °C overnight. The desired editing outcome was verified by colony PCR and subsequent sequencing.

### 2.7. Plasmid Curing

To cure both the pFnCpfAb-apr and the pCrAb-km plasmids in the desired mutant, the cells were inoculated into antibiotic-free LB broth medium and incubated at 37 °C overnight with shaking. Then, one loop of the bacterial suspension was streaked on an LB agar plate supplemented with 5% (*w*/*v*) sucrose and incubated at 37 °C overnight. Several colonies were picked up and streaked on LB agar plate with or without the supplementation of apramycin or kanamycin to confirm the successful curing. The cells with successful curing of both the pFnCpfAb-apr and the pCrAb-km plasmids could only grow on the antibiotic-free LB agar plate. The kanamycin should be replaced with the spectinomycin for the genome editing mediated by the pCrAb-spe plasmid.

### 2.8. Twitching Motility Assay

This assay was performed with modified LB agar motility plates (per liter, 5 g yeast extract, 5 g tryptone, 5 g NaCl, and 5 g agar [pH 7.2–7.4]). To test the twitching motility of the wild-type strain and the *pilT* deletion mutant, fresh overnight culture was picked with a sterile toothpick and stabbed to the boundary between the bottom of the agar layer and the polystyrene Petri dish. The inoculated plate was then immediately sealed with parafilm and incubated at 37 °C for 18 h. Representative images for each strain are shown.

### 2.9. Statistical Analyses

Each assay was repeated at least three times, and all values are represented as means ± standard deviation (SD). Unpaired two-tailed Student’s *t*-test was performed to evaluate statistical significance between groups using GraphPad Prism version 9.0.0. A *p*-value lower than 0.05 was considered statistically significant for all the assays.

## 3. Results

### 3.1. Construction of a CRISPR-FnCpf1-Based Two-Plasmid Genome Editing System in A. baumannii

**Figure 1:**
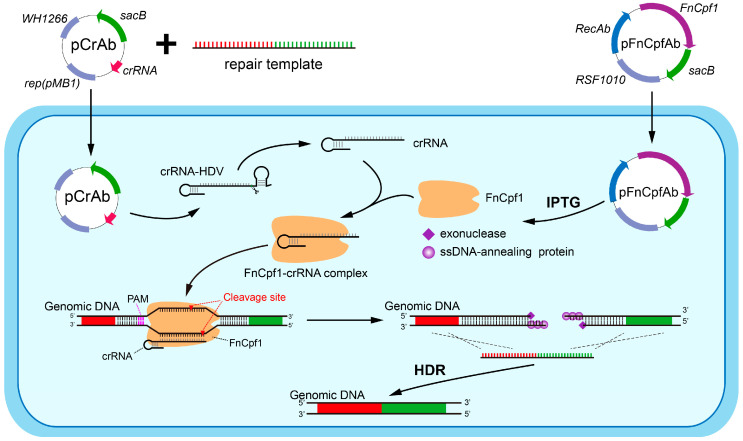
Schematic diagram of the procedures for CRISPR-FnCpf1-mediated genome-editing. The FnCpf1-crRNA complex recognizes and cleaves the target site and generates lethal double-strand break (DSB) in the genome of *A. baumannii*. The DSB is then repaired by the RecAb recombination system using a donor template, resulting in programmable genome modification.

**Figure 2:**
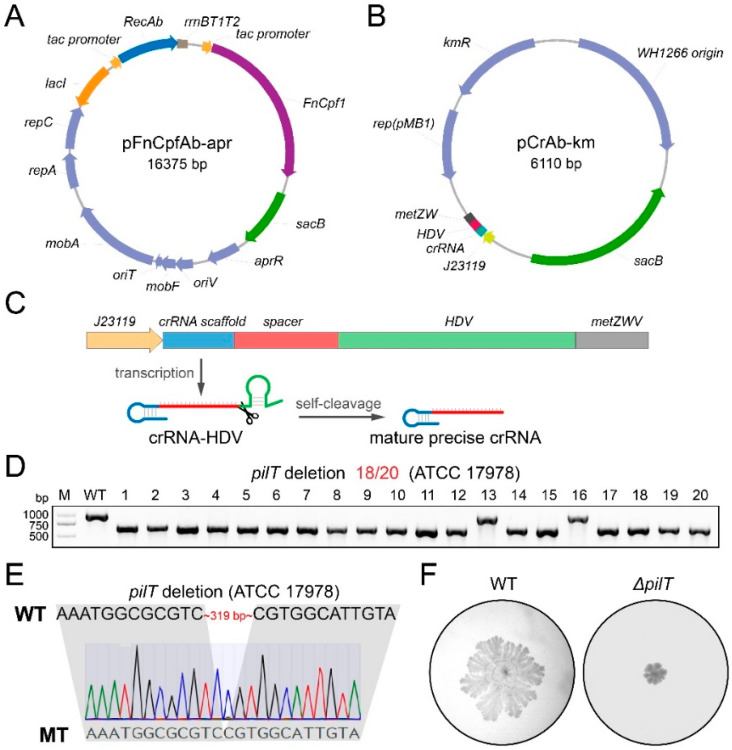
CRISPR-FnCpf1 coupled with the RecAb system enables efficient gene editing in *A. baumannii*. (**A**) Map of the pFnCpfAb-apr plasmid. The expressions of both the FnCpf1 nuclease and the RecAb recombination proteins are driven by the IPTG-inducible *tac* promoter. *aprR*—the apramycin resistance marker; *sacB*—sucrose-counter-selectable marker for plasmid curing after editing. (**B**) Map of the pCrAb-km plasmid. *J23119*, the promoter for crRNA expression; *HDV*, hepatitis delta virus ribozymes for precise RNA processing; *kmR*, the kanamycin resistance marker; and *sacB*, sucrose-counter-selectable marker for plasmid curing after editing. (**C**) Scheme of mature precise crRNA generation. The 3′-positioned HDV ribozymes mediate self-cleavage exactly to generate mature precise crRNA. (**D**) The *pilT* gene was successfully deleted with an efficiency of 18/20 in the *A. baumannii* ATCC 17978 strain, confirmed by PCR screening. M—Trans2K Plus II DNA Marker; WT—wild-type; 1–20—twenty randomly picked colonies. (**E**) The 317 bp within the *pilT* gene was deleted as expected, confirmed by Sanger sequencing. (**F**) The disruption of the *pilT* gene resulted in reduced twitching motility. WT—wild-type; Δ*pilT*—*pilT* deletion strain.

To harness CRISPR-FnCpf1 for genome editing in *A. baumannii*, we designed and constructed a two-plasmid system, including the pFnCpfAb-apr and the pCrAb-km plasmids (Figure 1). The pFnCpfAb-apr plasmid contains the apramycin resistance selection gene and the broad-host-range RSF1010 replicon [16], which is able to replicate in a wide range of Gram-negative bacteria including *E. coli* and *A. baumannii*. In addition, this plasmid expresses the LacI repressor protein, the synthetic codon-optimized FnCpf1 nuclease, and the exogenous RecAb recombination proteins, originated from *A. baumannii* IS-123 strain [17]. The expression of the LacI repressor is driven by a strong constitutive promoter, and the expression of both the FnCpf1 nuclease and the RecAb proteins is under the control of the LacI-repressible, IPTG-inducible promoter. The pCrAb-km plasmid possesses the kanamycin resistance selection gene and two independent replicons, ColE1 and WH1266, for replication in *E. coli* and *A. baumannii*, respectively [18] (Figure 2A). And the pCrAb-km plasmid contains the expression cassette for the fusion gene of crRNA-HDV (hepatitis delta virus) ribozyme with the constitutive *J23119* promoter and the *metZWV* terminator [19,20]. Two reversed *Bsa*I sites are inserted between the crRNA scaffold and the HDV ribozyme, enabling seamless insertion of spacers using Golden Gate assembly (Figure 2B). The crRNA-HDV fusion transcript can be further processed into the mature crRNA with precise 3′-end by the self-cleavage activity of the HDV ribozyme (Figure 2C). Moreover, the sucrose-sensitive *sacB* gene that works as a counter-selectable genetic marker is introduced into both the pFnCpfAb-apr and the pCrAb-km plasmids for easy plasmid curing after finishing the genome editing event [21].

To assess the capacity of the constructed two-plasmid system of genome editing in *A. baumannii*, we sought to delete the *pilT* gene, which encodes a protein that is required for retraction of the type IV pilus. We first electroporated the pFnCpfAb-apr plasmid into the *A. baumannii* ATCC 17978 strain to obtain the pFnCpfAb-apr-harboring cells. After the induction by 1 mM IPTG for 2 h, the cells that had expressed both the FnCpf1 nuclease and the RecAb recombination proteins were collected and prepared as the electrocompetent cells. Then, the pilT-spacer introduced pCrAb-km-pilT plasmid, which was co-transformed into the aforementioned electrocompetent cells with corresponding 80 nt ssODN template. After incubating at 37 °C overnight, as expected, several colonies were observed on the plate supplemented with apramycin and kanamycin. Twenty colonies were randomly selected to confirm the editing events, colony PCR showed that *pilT* deletion was achieved with an efficiency of 18/20 (Figure 2D), and further Sanger sequencing confirmed that these deleted sequences were as expected (Figure 2E). Because the deletion of the *pilT* gene renders the cells to reduce their motility [22], we also validated the inactivation of the *pilT* gene using a twitching motility assay (Figure 2F). Overall, these results indicated that the CRISPR-FnCpf1-based two-plasmid system possessed a great capacity for genome editing in *A. baumannii*.

The clinically isolated strains have diverse genetic backgrounds and are typically less amenable for genome editing. To expand the utility of CRISPR-FnCpf1 system, we tested the editing capacity of this system in two additional clinically isolated strains, ATCC 19606 and ABH6. Due to the inherent kanamycin resistance of the ABH6 strain, the kanamycin resistance gene in the pCrAb-km plasmid was replaced with the spectinomycin resistance gene through Gibson assembly, resulting in the pCrAb-spe plasmid. Following the above-mentioned method, we detected successful editing events for the *pilT* and the *pmrB* genes with high efficiency in both the ATCC 19606 and ABH6 strains. The editing efficiencies of *pilT* and *pmrB* were 17/20 (Figure S2A) and 20/20 (Figure S2B), respectively, in the ATCC 19606 strain. Additionally, the editing efficiencies were 15/20 for *pilT* (Figure S2C) and 19/20 for *pmrB* (Figure S2D) in the ABH6 strain. Together, these results indicated that the CRISPR-FnCpf1 system was an effective and powerful tool in various *A. baumannii* strains.

The editing plasmids that existed in the desired mutants may have interference with subsequent physiological, genetic, metabolic, and pathogenic research. As a proof of principle, we attempted to eliminate both the pFnCpfAb-apr and the pCrAb-km plasmids in the *pilT* deletion mutant. To do this, a single colony harboring the two plasmids was inoculated into 5 mL of antibiotic-free LB medium and grown with shaking overnight at 37 °C. Next, a loopful culture was streaked onto an LB plate in the presence of 5% (*w*/*v*) sucrose at 37 °C until colonies were visible. Four individual colonies from the sucrose plate were randomly picked and streaked onto three different LB plates supplemented with apramycin or kanamycin or without antibiotics. As shown in Figure S3, all the four colonies only grew normally on the antibiotic-free plate, whereas no growth of colonies was observed on the plates with apramycin or kanamycin, confirming that both of the two plasmids could be efficiently cured after successful editing by the *sacB* counter-selection on 5% (*w*/*v*) sucrose plate.

### 3.2. Determination of the FnCpf1 PAM Sequence and Spacer Length in A. baumannii

**Figure 3:**
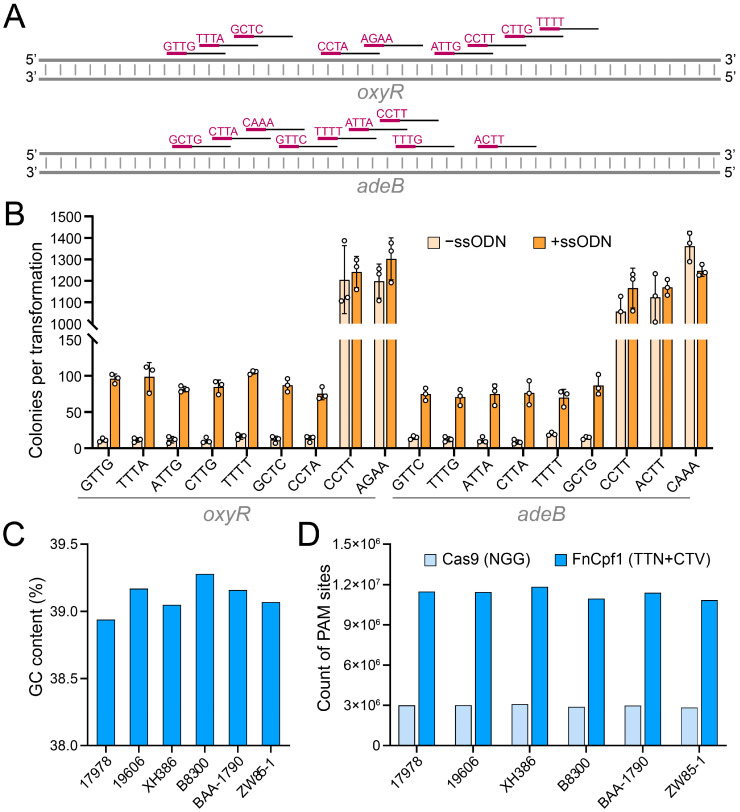
PAM requirement of FnCpf1-mediated gene editing in *A. baumannii*. (**A**) Scheme of spacer sites with different PAMs in the *oxyR* and the *adeB* genes. PAM sequences and spacer sites are in purple and black, respectively. (**B**) The colonies produced by electroporating the spacer-introduced pCrAb-km plasmids that target various 5′-NYTN-3′ PAMs into the IPTG-induced pFnCpfAb-apr-harboring *A. baumannii* cells. The 5′-AGAA-3′ and the 5′-CAAA-3′ PAMs were used as control. 1/10 of the transformed cells, either with plasmid and ssODN or with only plasmid, were plated. Data were means ± SD (n = 3). (**C**) GC contents of the *A. baumannii* strains ATCC 17978 (GenBank: CP000521), ATCC 19606 (GenBank: NZ_CP046654), XH386 (GenBank: NZ_CP021326), B8300 (GenBank: CP021347), ATCC BAA-1790 (GenBank: NZ_CP042841), and ZW85-1 (GenBank: CP006768). (**D**) The targetable PAM count of Cas9 and FnCpf1 in various *A. baumannii* strains. The 5′-NGG-3′ are used as PAMs for Cas9. The 5′-TTN-3′ and 5′-CTV-3′ are used as PAMs for FnCpf1.

The PAM sequence located at the 5′ end of the target sequence is essential for efficient targeting by FnCpf1 [23]. However, it appears that the PAM sequence that was recognized efficiently by FnCpf1 seems to be not completely identical in different species. For instance, Zetsche et al. reported that the PAM requirement for FnCpf1 was the 5′-TTN-3′ (N = A, T, C or G) in *E. coli* by plasmid depletion assay [11]. Tu et al. revealed that the requirements for a FnCpf1 PAM sequence in human cells was the 5′-KYTV-3′ (K = G or T, Y = C or T, V = A, C or G) [24]. Wang et al. demonstrated that FnCpf1 preferred to recognize the 5′-KTTV-3′ in *S. aureus* [25]. In order to determine the optimal PAM sequences for FnCpf1 in *A. baumannii*, we evaluated the genomic cleavage and recombination editing efficiency by targeting the *oxyR* and the *adeB* genes with various 5′-NYTN-3′ PAMs (Figure 3A). Due to the absence of intrinsic non-homologous end joining (NHEJ) and homologous recombination (HR) repair pathways, a successful targeting event could kill *A. baumannii* cells through the DSB that is generated by FnCpf1. Only the cells that have repaired the lethal genomic DSB through RecAb recombination system with ssODN can survive. The results showed that the transformation of target sites with the 5′-NTTN-3′ and the 5′-NCTV-3′ PAMs yielded significantly fewer colonies than that of other PAMs (Figure 3B), indicating that the PAM requirement for efficient cleavage of target sites by FnCpf1 is the 5′-NTTN-3′ and the 5′-NCTV-3′ in *A. baumannii*.

Considering the low GC content of the *A. baumannii* genome, we selected six strains with complete genome sequences from the GenBank database to calculate their GC content (Figure 3C) and the number of PAM sites for FnCpf1 and Cas9 (with a 5′-NGG-3′ PAM), respectively. As shown in Figure 3D, the number of FnCpf1 PAM sites is nearly four times higher than that of Cas9 in each strain, suggesting that genome editing with FnCpf1 provides greater target site selectivity and flexibility in *A. baumannii*.

**Figure 4:**
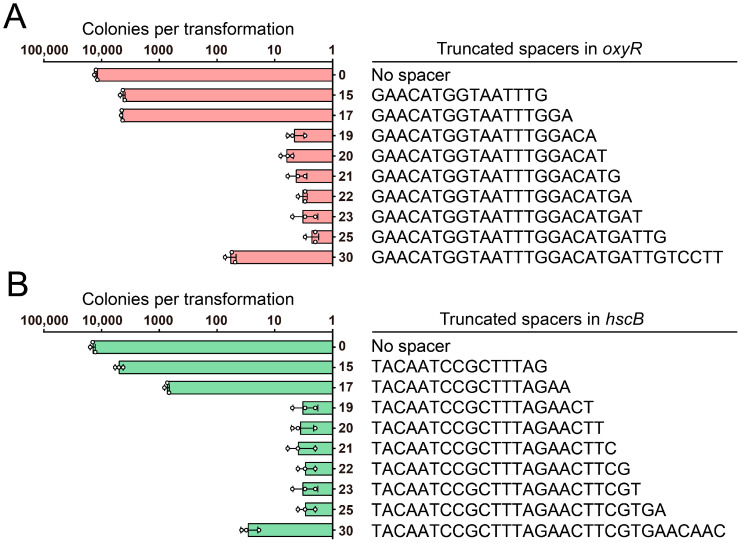
Spacer length requirement of FnCpf1-mediated gene editing in *A. baumannii*. The colonies produced by electroporating the pCrAb-km plasmids containing various truncated spacers into the IPTG-induced pFnCpfAb-apr-harboring *A. baumannii* cells to target the *oxyR* gene (**A**) and the *hscB* gene (**B**), respectively. The pCrAb-km plasmid containing no spacer was used as control. Data were means ± SD (n = 3).

The targeting efficiency of FnCpf1 is also influenced by the length of the spacer. To investigate the influence of the spacer length on the cleavage efficiency of FnCpf1 in *A. baumannii*, we constructed and tested a series of crRNAs with different spacer lengths (15, 17, 19, 20, 21, 22, 23, 25, 30 nt) to target a site in the *oxyR* gene. Due to the lethality of DSBs that are generated by CRISPR-FnCpf1 cleaving genomic DNA in *A. baumannii*, a lower number of colonies under the same transformation conditions indicates a higher efficiency of genomic DNA cleavage. As shown in Figure 4A, fewer than 10 colonies were observed for the transformation mediated by spacers with lengths ranging from 19 to 25 nt, whereas approximately 10^3^ to 10^4^ colonies could be obtained for the same transformation using spacers with other lengths or an empty spacer. This observation was further confirmed at another site in the *hscB* gene, and similar results were obtained (Figure 4B). Taken together, these results demonstrated that a spacer for FnCpf1 with a length ranging from 19 to 25 nt could lead to efficient genome cleavage in *A. baumannii*.

### 3.3. Comparison of Cas9 and FnCpf1 Editing Performance in A. baumannii

The CRISPR-Cas9 system has been successfully applied for genome editing in *A. baumannii* [5]. However, sometimes, the number of colonies after editing by Cas9 was low, especially in some clinically isolated strains, which may be attributed to the inhibitory effect of the blunt ends that are generated by Cas9 on recombinational repair by RecAb [13] or the cellular toxicity of Cas9 [26]. Different from Cas9, FnCpf1 generates sticky ends with a 5′ overhang after cleavage, which could potentially result in the generation of more colonies. Moreover, FnCpf1 has been reported to cause less toxic side effects in bacteria than Cas9 [27].

**Figure 5:**
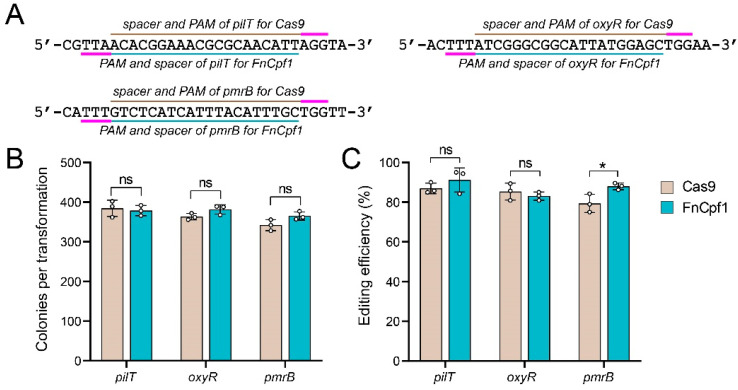
Comparison of Cas9 and FnCpf1 editing performance at three identical sites in the *A. baumannii* ATCC 17978 strain. (**A**) Three target sites, located in *pilT*, *oxyR*, and *pmrB*, harbor the canonical PAMs of Cas9 and FnCpf1 on both ends. (**B**) The FnCpf1 system yielded a similar number of colonies as the Cas9 system when using the same spacer to edit the same region. Data were means ± SD (n = 3). Two-tailed Student’s *t*-test was used for statistical analysis. (**C**) Cas9 and FnCpf1 systems exhibited similar editing efficiencies at the *pilT* and the *oxyR* loci; however, FnCpf1 system exhibited slightly higher editing efficiency at the *pmrB* locus when using the same spacer to edit the same region. Data were means ± SD (n = 3). Two-tailed Student’s *t*-test was used for statistical analysis (ns, no significance; * *p* < 0.05).

To compare the editing performance of Cas9 and FnCpf1, we tested three identical target sequences, including in *pilT*, *oxyR*, and *pmrB*, harboring the canonical PAMs on both ends of the target sequences (Figure 5A). Equimolar sgRNA or crRNA expression plasmid was transformed individually into the IPTG-induced pCasAb-apr or pFnCpfAb-apr-harboring *A. baumannii* ATCC 17978 cells with identical 80 nt ssODN (40 nt each). At all three sites, similar colony counts were observed following the transformation of Cas9 and FnCpf1 (Figure 5B). Furthermore, at the *pilT* and the *oxyR* sites, the editing efficiency of Cas9 and FnCpf1 was similar, except at the *pmrB* site, where FnCpf1 had slightly higher editing efficiency than Cas9 (Figure 5C). To further confirm the editing performance, we performed the same experiment in the ABH6 strain and obtained similar results (Figure S4), thus suggesting that FnCpf1 and Cas9 have comparable editing performance in *A. baumannii*.

### 3.4. Large-Fragment Deletion by CRISPR-FnCpf1 in A. baumannii

Gene clusters play crucial roles in various biological processes in *A. baumannii*, such as drug resistance, pathogenicity islands, and biofilm promotion. In order to study the function of gene clusters, it is an important step to delete them. However, gene clusters, composed of large DNA fragments, are often challenging to delete using a single sgRNA with the CRISPR-Cas9 tool, and because the distance between homologous arms and DSB sites generated by Cas9 is too far, it can hinder subsequent recombination repair. Some studies showed that using dual sgRNAs targeting two ends of the editing region could delete large DNA fragments [28,29], but the construction of two sgRNA expression cassettes was more complex and time-consuming.

**Figure 6:**
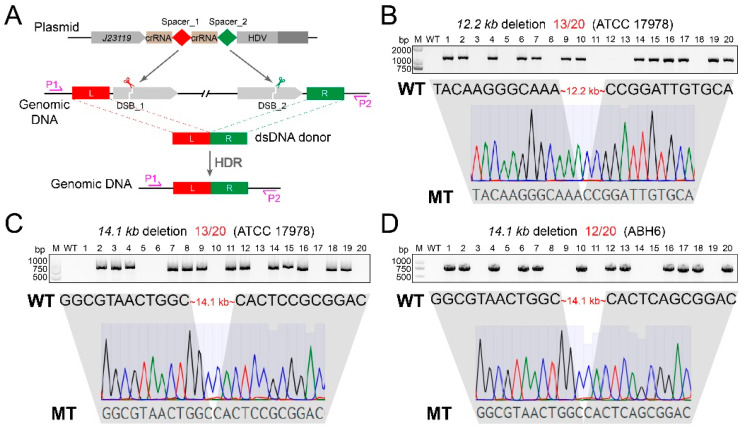
CRISPR-FnCpf1-mediated large-fragment deletion in *A. baumannii*. (**A**) Schematic diagram of CRISPR-FnCpf1-mediated large-fragment deletion through the strategy of dual crRNAs in tandem. Two DSBs are generated at each end of the large-fragment region, and then repaired by homology-dependent recombination (HDR) using a dsDNA donor template. (**B**) The 12.2 kb fragment was deleted with an efficiency of 13/20 in the *A. baumannii* ATCC 17978 strain, confirmed by PCR screening and Sanger sequencing. (**C**) The 14.1 kb fragment of the ATCC 17978 strain was deleted with an efficiency of 13/20. (**D**) The 14.1 kb fragment of the ABH6 strain was deleted with an efficiency of 12/20. M—Trans2K Plus II DNA Marker; WT—wild-type; 1–20—twenty randomly picked colonies.

Given the capacity of FnCpf1 to generate multiple mature crRNAs from a single concatemeric crRNA precursor [15], we assembled a crRNA expression cassette with two spacers, *J23119*-crRNA-spacer_1-crRNA-spacer_2-HDV-*metZWV*, by performing a single Golden Gate assembly reaction. As an example, spacer_1 and spacer_2 were designed to target both ends of the approximately 12.2 kb region. Then, the two-spacer-introduced pCrAb-km plasmid was co-transformed into the IPTG-induced pFnCpfAb-apr-harboring *A. baumannii* cells with 80 nt ssODN (40 nt each) to delete the 12.2 kb fragment in the ATCC 17978 strain (Figure 6A). The transformation of only the two-spacer-introduced pCrAb-km plasmid without ssODN was used as control. Unfortunately, fewer than 10 colonies were observed for each transformation, regardless of the presence or absence of ssODN. Further PCR screening revealed that none of them were the desired 12.2 kb fragment deletion mutants, indicating that the ssODN was insufficient for large-fragment deletion repair in *A. baumannii*.

Considering that the use of dsDNA repair templates can generate more colonies [30], we assessed the ability of plasmid-borne dsDNA templates and linear dsDNA templates to achieve the 12.2 kb fragment deletion. The transformation of pCrAb-km containing both two spacers and 800 bp dsDNA donor template yielded approximately 200 colonies with an efficiency of 13/20, confirmed by PCR screening and Sanger sequencing (Figure 6B). In addition, approximately 50 colonies were observed for the co-transformation of the two-spacer-introduced pCrAb-km plasmid and linear 800 bp dsDNA donor, and the deletion efficiency was 7/20 (Figure S5). Similarly, we also successfully deleted the A1S_0112-A1S_0119 gene cluster (~14.1 kb) that contains eight genes that are responsible for biofilm formation and virulence by using the dual sgRNAs and plasmid-borne dsDNA template in both the ATCC 17978 strain and the clinically isolated ABH6 strain [31], and the efficiencies were 13/20 (Figure 6C) and 12/20 (Figure 6D), respectively. Taken together, these results demonstrated that the CRISPR-FnCpf1 tool can perform large-fragment deletion coupled with dual sgRNAs and dsDNA donor template in *A. baumannii*.

### 3.5. Multiplex Gene Editing by CRISPR-FnCpf1 in A. baumannii

Multiplex gene editing with CRISPR-Cas9 tool is often limited by the requirement for multiple sgRNA cassette constructs or time-consuming rounds of single-gene editing. However, FnCpf1 offers a more efficient approach by easily maturing multiple crRNAs using a single crRNA array [15], suggesting that the CRISPR-FnCpf1 tool may be amenable to simultaneous multiplex editing in *A. baumannii*.

**Figure 7:**
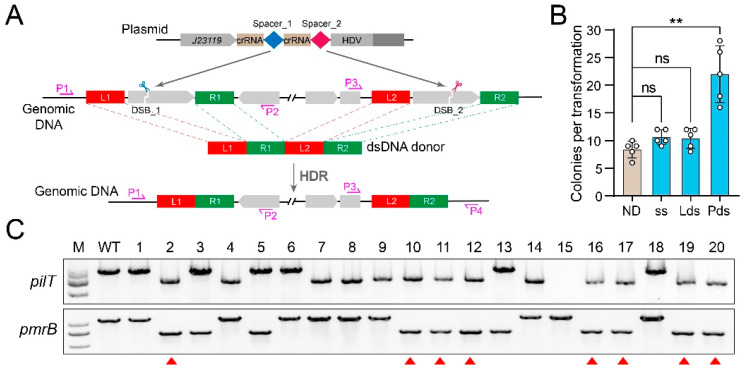
CRISPR-FnCpf1-mediated multiplex gene editing in *A. baumannii*. (**A**) Schematic diagram of CRISPR-FnCpf1-mediated multiplex gene editing. Two distinct spacers guide the corresponding crRNA-FnCpf1 complex to cleave their respective target sites, and then two DSBs are repaired individually by the HDR using a tandem dsDNA donor template. (**B**) The multiplex editing coupled with the plasmid-borne dsDNA template yielded more colonies than that with other donor templates. ND—No donor, ss—ssODN, Lds—Linear dsDNA donor, Pds—Plasmid-borne dsDNA donor. Data were means ± SD (n = 5). Two-tailed Student’s *t*-test was used for statistical analysis (ns, no significance; ** *p* < 0.01). (**C**) The *pilT* and the *pmrB* genes were simultaneously deleted with an efficiency of 8/20, confirmed by PCR screening. The red triangles represent successful double-gene deletion colonies. M—Trans2K Plus II DNA Marker; WT—wild-type; 1–20—twenty randomly picked colonies.

To investigate the multiplex editing ability of the CRISPR-FnCpf1 tool, the *J23119*-crRNA_pilT-crRNA_pmrB-HDV-*metZWV* expression cassette was assembled to target the *pilT* gene and the *pmrB* gene simultaneously (Figure 7A). We tested three types of donor DNA, including a mixture of ssODN_pilT and ssODN_pmrB, linear dsDNA, and plasmid-borne dsDNA, as repair templates. After transformation of the two-spacer-introduced pCrAb-km plasmid and the corresponding donor DNA, different colony numbers were observed. As shown in Figure 7B, only the transformation with the plasmid-borne dsDNA repair template resulted in a significant increase in colony numbers, while the transformation with linear dsDNA or ssODN showed no significant difference in colony numbers compared to that of the no repair template (Figure 7B). Two pairs of primers covering the editing regions were designed and used to verify the simultaneous editing events. Further PCR screening showed that out of 20 randomly selected colonies, 8/20 colonies achieved simultaneous editing of the *pilT* and *pmrB* genes (Figure 7C), manifesting the capacity for multiplex gene editing in *A. baumannii*.

## 4. Discussion

In this study, we have engineered a CRISPR-FnCpf1-based two-plasmid system, including the pFnCpfAb and the pCrAb, for convenient and precise genome editing in *A. baumannii*. Our results indicated that for achieving efficient genome editing in *A. baumannii*, the PAM sequence of the 5′-TTN-3′ and the 5′-CTV-3′ and a length of spacer ranging from 19 to 25 nt were preferred for this system.

Although the CRISPR-Cas9 system has been reported to carry out various genome manipulations in *A. baumannii*, the CRISPR-FnCpf1 system still has advantages over the CRISPR-Cas9 system [5]. For example, large-fragment deletion and multiplex gene editing remain challenging for the CRISPR-Cas9 system, requiring complex and time-consuming plasmid construction or multiple rounds of editing. Fortunately, FnCpf1 has been reported to have the capacity to process multiple mature crRNAs using a single crRNA array [15]. With this feature, we have achieved large-fragment deletion and multiple gene editing using a tandem multiple crRNA expression cassette, which can be assembled easily through a single Golden Gate assembly reaction.

In studies of gene function related to antibiotic resistance or virulence, it is sometimes necessary to inactivate multiple genes. For example, when identifying the function of three β-lactamase genes, *blaTEM-1D*, *blaADC-25*, and *blaOXA-23*, which are associated with imipenem and sulbactam resistance in the *A. baumannii* XH386 strain [5], using the newly developed FnCpf1-based tool for simultaneous inactivation of all the three genes would be more time-saving and simpler compared to the Cas9-based tool, which requires three rounds of genetic manipulation.

ssODN, which can be conveniently synthesized, has been proven to be useful as a repair template for gene editing in various bacteria, including *A. baumannii* [5]. However, we found that ssODN was ineffective for large-fragment deletion and multiplex editing. Furthermore, we also revealed that only the plasmid-borne dsDNA donor, not the linear dsDNA donor, could be used as repair templates for multiplex editing. The possible reason for these situations may be that the recombination repair ability of the RecAb proteins that are originated from the *A. baumannii* IS-123 strain is not sufficiently amenable when repairing two or more DSBs with ssODN. Therefore, it remains valuable to search for new superior recombination systems, similar to lambda-Red and Rac-RecET [32,33], in the bacteriophages of *A. baumannii* to enhance genetic manipulation.

Cas9 creates blunt ends after cleavage, while Cpf1 generates sticky ends with 5’-overhang, which may increase the efficiency of the homologous recombination. Additionally, some studies have reported that the cellular toxicity of FnCpf1 is much lower than that of Cas9. However, no significant improvement in transformation efficiency or editing efficiency mediated by the FnCpf1 was observed compared to the Cas9 in *A. baumannii*. Both Cas9 and FnCpf1 exhibited similar editing performance in *A. baumannii*. Nevertheless, due to the low GC content of the genome, FnCpf1 offers a wider selection of the cleavage targets in *A. baumannii*, which is particularly important for point mutations or labeling specific gene loci.

Additionally, the development of innovative base editors in *A. baumannii* is theoretically feasible by fusing cytidine deaminase enzyme or adenine deaminase enzyme with a nuclease-inactivated version of FnCpf1 (dFnCpf1) [34,35]. Notably, these dFnCpf1-based base editors can achieve C to T or A to G conversions at different locations compared to dCas9 or Cas9 nickase-based base editors due to the distinct PAM sequences of FnCpf1 and Cas9. By combining the use of these two types of base editors, we can significantly expand the scope of base editing in the genome of *A. baumannii*, contributing to a range of valuable applied research in the future.

## 5. Conclusions

In this study, we have successfully developed a two-plasmid CRISPR-FnCpf1 tool for precise and versatile genome editing in *A. baumannii*. This newly developed tool prefers to recognize the 5′-TTN-3′ (N = A, T, C or G) and the 5′-CTV-3′ (V = A, C or G) PAM sequence and utilizes spacers ranging from 19 to 25 nt in *A. baumannii*. Compared to the existing CRISPR-Cas9 tool, the CRISPR-FnCpf1 tool has similar editing capabilities but offers approximately four times the targetable range in the genome of *A. baumannii*. Furthermore, we have demonstrated its effectiveness in complementing the CRISPR-Cas9 system, enabling large-fragment deletion and multiplex editing capabilities through a tandem dual crRNA expression cassette and dsDNA donor template. Therefore, this new system is highly valuable and can significantly expand the genome editing toolbox for *A. baumannii*. By combining the use of these diverse genome editing tools, we can significantly advance genetic engineering in *A. baumannii*, facilitating a wide range of investigations into pathogenesis, antibiotic resistance mechanisms, and therapeutic development.

## Data Availability

The plasmids constructed in this study have been deposited in Addgene with the accession codes 214198 for pFnCpfAb-apr and 214199 for pCrAb-km.

## Supplementary Materials

The following supporting information can be downloaded at https://www.mdpi.com/article/10.3390/cimb46010037/s1: Figure S1: Map of the pKOBEG-rpsL-FnCpf1 plasmid. Figure S2: Gene deletion in clinically isolated *A. baumannii* strains. Figure S3: Results of plasmid curing. Figure S4: Comparison of Cas9 and FnCpf1 editing performance at three identical sites in the *A. baumannii* ABH6 strain. Figure S5: The 12.2 kb fragment was deleted with an efficiency of 13/20 when using the linear dsDNA donor as repair template in the *A. baumannii* ATCC 17978 strain, confirmed by PCR screening. Table S1: Plasmids used in this study. Table S2: Primers and ssODN donors used in this study.
