# Supplementary material for: The Development of a CRISPR-FnCpf1 System for Large-Fragment Deletion and Multiplex Gene Editing in Acinetobacter baumannii
Source: Curr Issues Mol Biol. 2024 Jan 5;46(1):570–84. doi: 10.3390/cimb46010037 (PMC10814444; doi:10.3390/cimb46010037)
Supplement: Supplementary file 1 [file cimb-46-00037-s001.zip › cimb-2797633-supplementary.pdf]

Supplementary Materials for

The Development of a CRISPR-FnCpf1 System for Large-Fragment Deletion and Multiplex Gene Editing in *Acinetobacter baumannii*

Shuai Wang [1,2], Yue Ding [1,2], Hua Rong [1,2,*], and Yu Wang [1,2,*]

1 College of Bioscience and Bioengineering, Jiangxi Agricultural University, Nanchang 330045, China
wangshuai98125@163.com (S.W.); dyueyue0712@163.com (Y.D.)

2 Nanchang City Key Laboratory of Animal Virus and Genetic Engineering, Nanchang 330045, China

* Correspondence: ronghua@jxau.edu.cn (H.R.); wangyu@jxau.edu.cn (Y.W.)

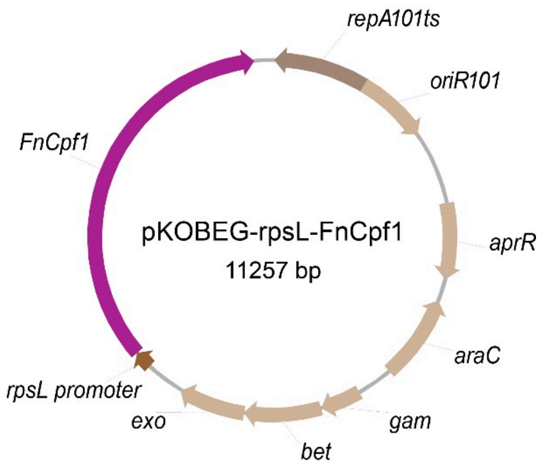

Figure S1. Map of the pKOBEG-rpsL-FnCpf1 plasmid. The FnCpf1 coding fragment was amplified by PCR from this plasmid in this study.

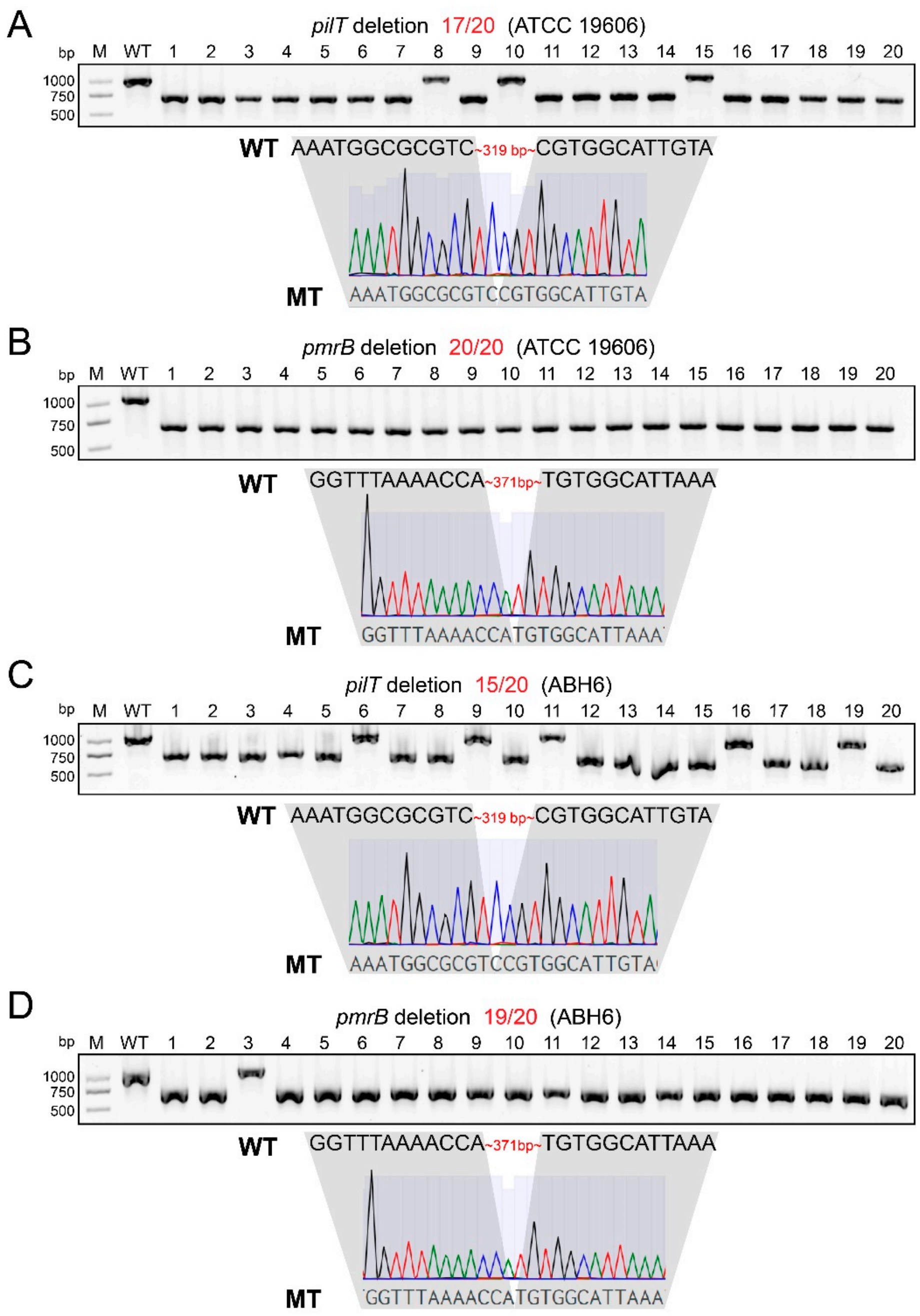

Figure S2. Gene deletion in clinically isolated *A. baumannii* strains. (A) The *pilT* gene was deleted with an efficiency of 17/20 in the *A. baumannii* ATCC 19606 strain. (B) The *pmrB* gene was deleted with an efficiency of 20/20 in the *A. baumannii* ATCC 19606 strain. (C) The *pilT* gene was deleted with an efficiency of 15/20 in the *A. baumannii* ABH6 strain. (D) The *pmrB* gene was deleted with an efficiency of 19/20 in the *A. baumannii* ABH6 strain. M—Trans2K Plus II DNA Marker; WT—wild-type; 1–20—twenty randomly picked colonies.

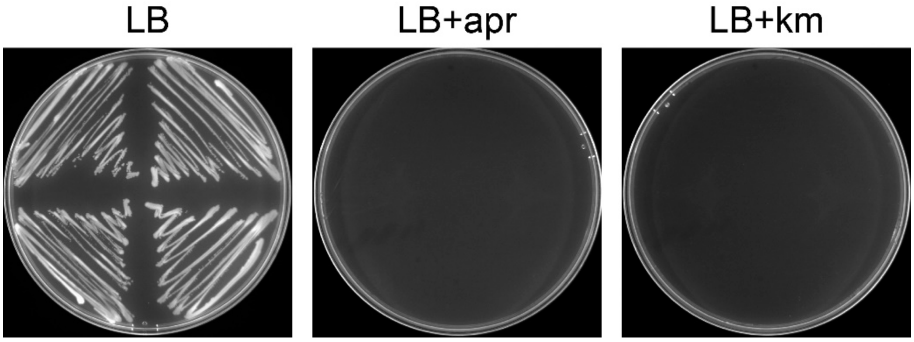

Figure S3. Results of plasmids curing. Both the pFnCpfAb-apr and the pCrAb-km plasmids could be easily cured simultaneously by culturing the cells in an antibiotic-free LB medium overnight and then streaking them onto the LB agar plate containing 5% (*w*/*v*) sucrose. apr, apramycin; km, kanamycin.

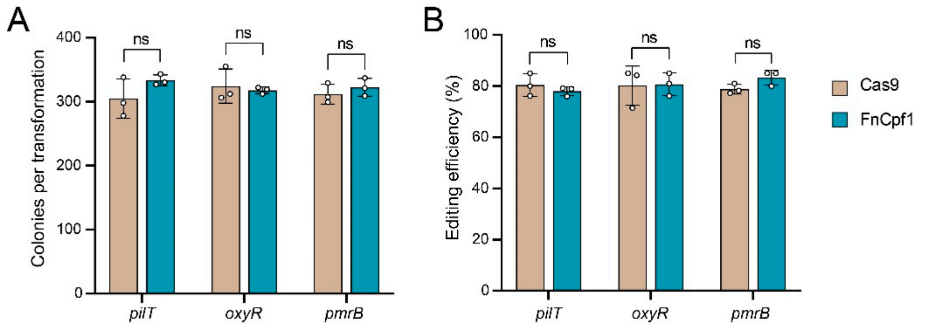

Figure S4. Comparison of Cas9 and FnCpf1 editing performance at three identical sites in the *A. baumannii* ABH6 strain. (A) The FnCpf1 system yielded a similar number of colonies as the Cas9 system when using the same spacer to edit the same region. Data were means ± SD (n = 3). Two-tailed Student's *t*-test was used for statistical analysis. (B) Cas9 and FnCpf1 systems exhibited similar editing efficiencies at three loci when using the same spacer to edit the same region. Data were means ± SD (n = 3). Two-tailed Student's *t*-test was used for statistical analysis (ns, no significance).

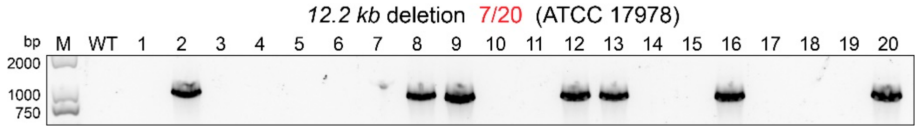

Figure S5. The 12.2 kb fragment was deleted with an efficiency of 13/20 when using the linear dsDNA donor as repair template in the *A. baumannii* ATCC 17978 strain, confirmed by PCR screening. M—Trans2K Plus II DNA Marker; WT—wild-type; 1–20—twenty randomly picked colonies.
