# Supplementary figures and images for: The Development of a CRISPR-FnCpf1 System for Large-Fragment Deletion and Multiplex Gene Editing in Acinetobacter baumannii
Source: Curr Issues Mol Biol. 2024 Jan 5;46(1):570–84. doi: 10.3390/cimb46010037 (PMC10814444; doi:10.3390/cimb46010037)

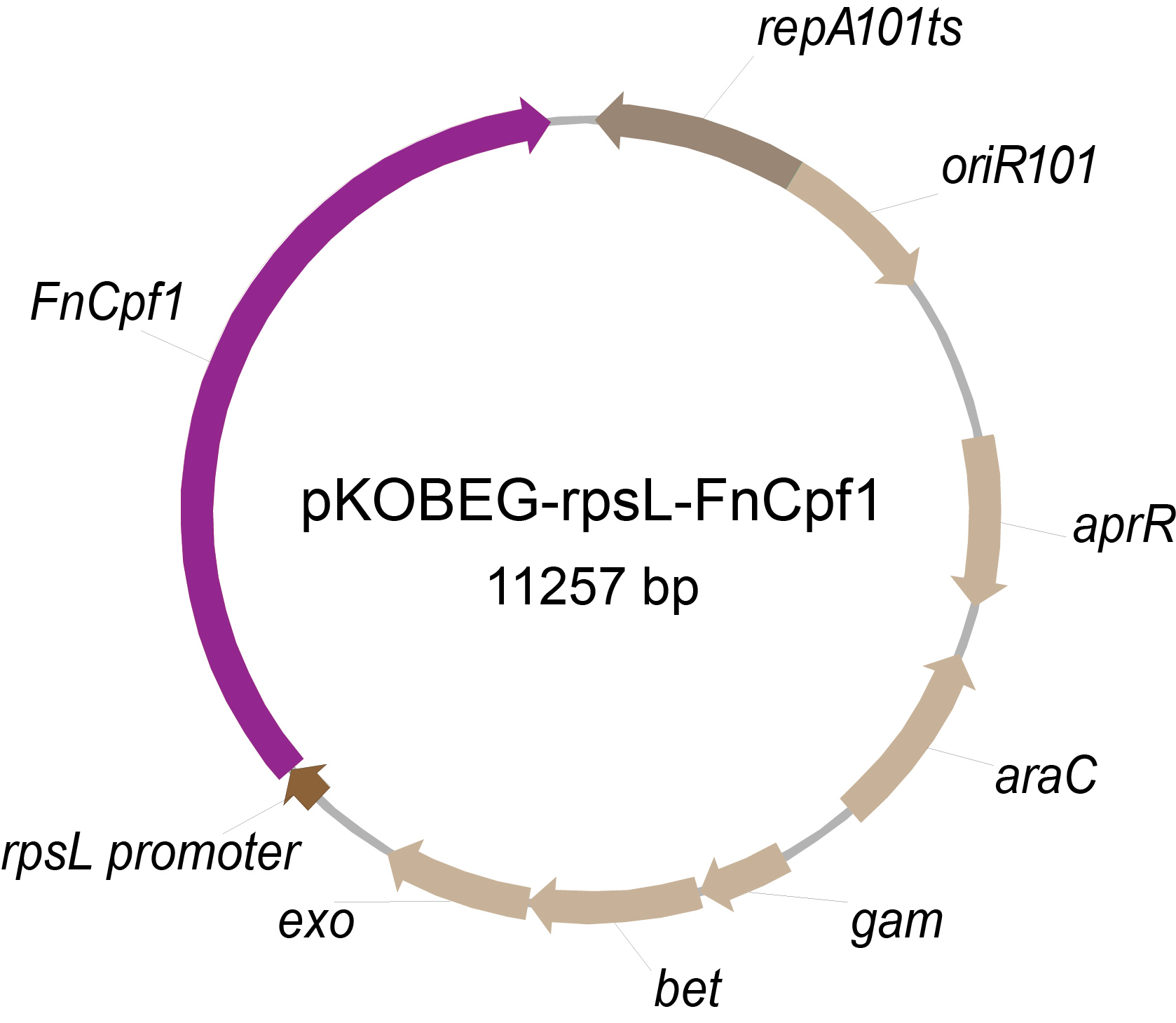

Supplement: Supplementary file 1 [file cimb-46-00037-s001.zip › Figure S1.jpg]

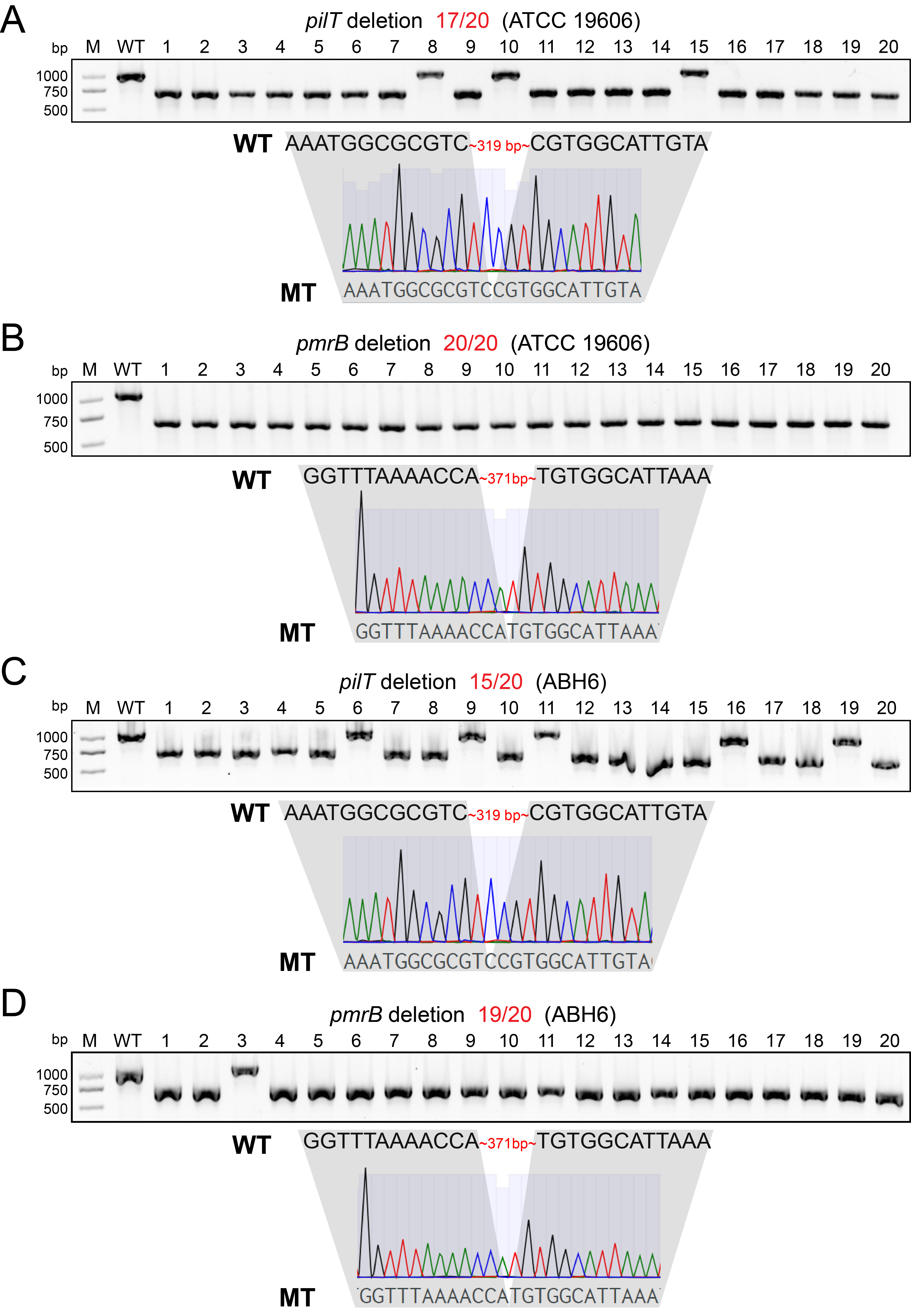

Supplement: Supplementary file 1 [file cimb-46-00037-s001.zip › Figure S2.jpg]

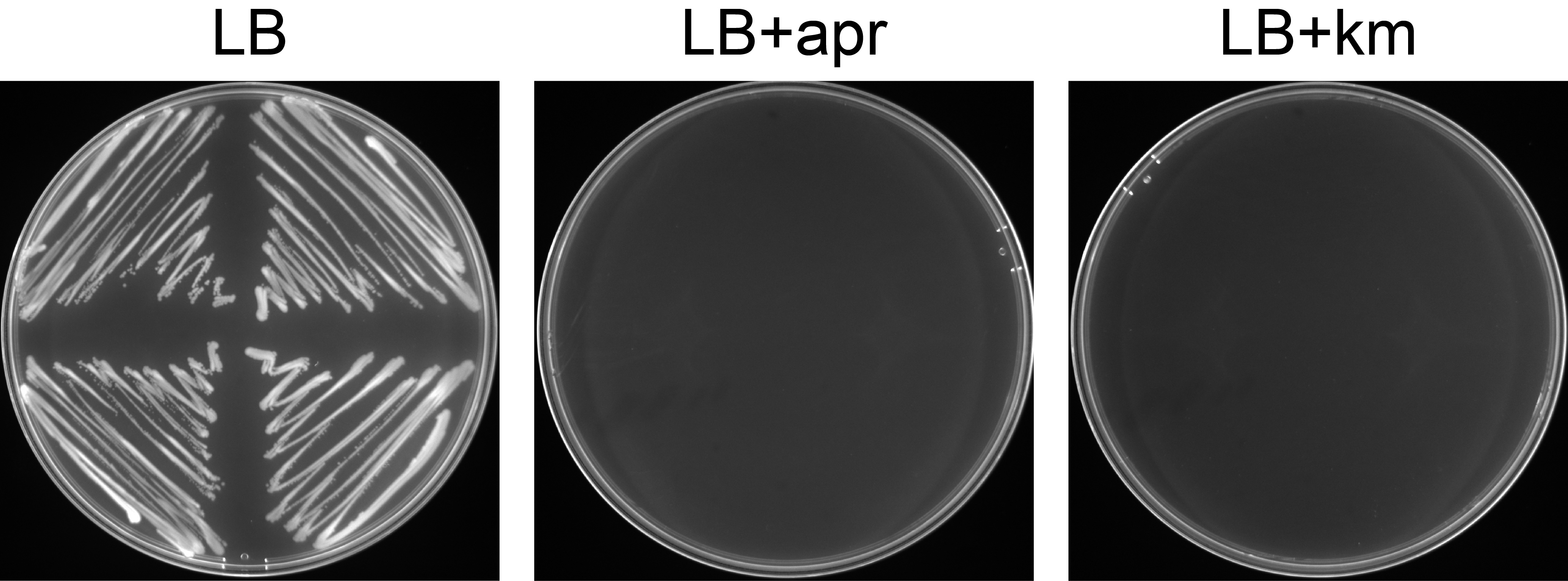

Supplement: Supplementary file 1 [file cimb-46-00037-s001.zip › Figure S3.jpg]

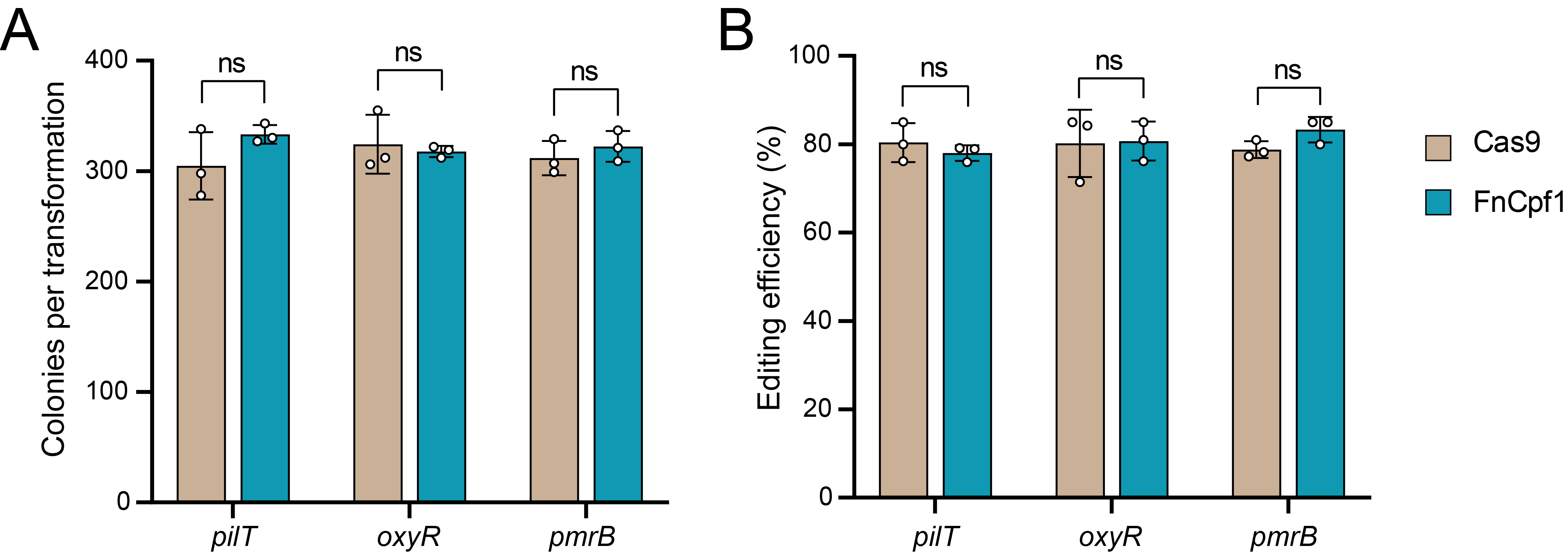

Supplement: Supplementary file 1 [file cimb-46-00037-s001.zip › Figure S4.jpg]

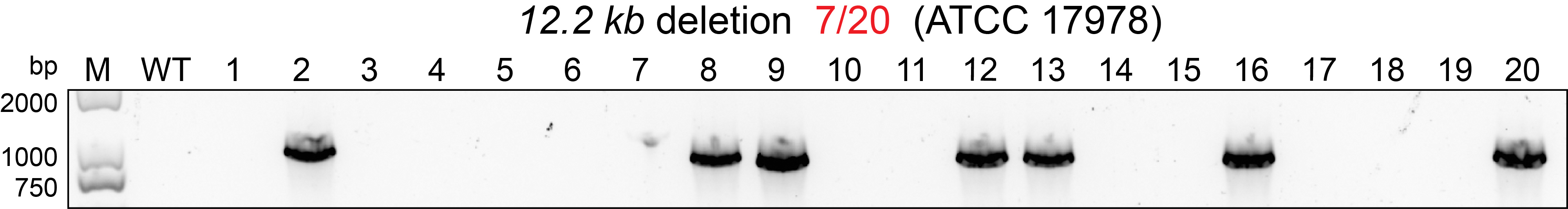

Supplement: Supplementary file 1 [file cimb-46-00037-s001.zip › Figure S5.jpg]
